# Killing of diverse eye pathogens (*Acanthamoeba spp*., *Fusarium solani*, and *Chlamydia trachomatis*) with alcohols

**DOI:** 10.1371/journal.pntd.0005382

**Published:** 2017-02-09

**Authors:** Yousuf Aqeel, Raquel Rodriguez, Aparajita Chatterjee, Robin R. Ingalls, John Samuelson

**Affiliations:** 1 Department of Molecular and Cell Biology, Boston University Goldman School of Dental Medicine, Boston, Massachusetts, United States of America; 2 Department of Medicine, Section of Infectious Diseases, Boston Medical Center, Boston, Massachusetts, United States of America; 3 Department of Microbiology, Boston University School of Medicine, Boston, Massachusetts, United States of America; Fondation Raoul Follereau, FRANCE

## Abstract

**Background:**

Blindness is caused by eye pathogens that include a free-living protist (*Acanthamoeba castellanii*, *A*. *byersi*, and/or other *Acanthamoeba spp*.), a fungus (*Fusarium solani*), and a bacterium (*Chlamydia trachomatis*). Hand-eye contact is likely a contributor to the spread of these pathogens, and so hand washing with soap and water or alcohol–based hand sanitizers (when water is not available) might reduce their transmission. Recently we showed that ethanol and isopropanol in concentrations present in hand sanitizers kill walled cysts of *Giardia* and *Entamoeba*, causes of diarrhea and dysentery, respectively. The goal here was to determine whether these alcohols might kill infectious forms of representative eye pathogens (trophozoites and cysts of *Acanthamoeba*, conidia of *F*. *solani*, or elementary bodies of *C*. *trachomatis*).

**Methodology/Principal findings:**

We found that treatment with 63% ethanol or 63% isopropanol kills >99% of *Acanthamoeba* trophozoites after 30 sec exposure, as shown by labeling with propidium iodide (PI) and failure to grow in culture. In contrast, *Acanthamoeba* cysts, which contain cellulose fibers in their wall, are relatively more resistant to these alcohols, particularly isopropanol. Depending upon the strain tested, 80 to 99% of *Acanthamoeba* cysts were killed by 63% ethanol after 2 min and 95 to 99% were killed by 80% ethanol after 30 sec, as shown by PI labeling and reduced rates of excystation *in vitro*. Both ethanol and isopropanol (63% for 30 sec) kill >99% of *F*. *solani* conidia, which have a wall of chitin and glucan fibrils, as demonstrated by PI labeling and colony counts on nutrient agar plates. Both ethanol and isopropanol (63% for 60 sec) inactivate 96 to 99% of elementary bodies of *C*. *trachomatis*, which have a wall of lipopolysaccharide but lack peptidoglycan, as measured by quantitative cultures to calculate inclusion forming units.

**Conclusions/Significance:**

In summary, alcohols kill infectious forms of *Acanthamoeba*, *F*. *solani*, and *C*. *trachomatis*, although longer times and higher ethanol concentrations are necessary for *Acanthamoeba* cysts. These results suggest the possibility that expanded use of alcohol-based hand sanitizers in places where water is not easily available might reduce transmission of these important causes of blindness.

## Author summary

Hand washing with soap and water is an important public health tool for reducing transmission of viruses, bacteria, fungi, and protists. Alcohol-based hand sanitizers, which are widely dispensed in hospitals and public places, kill many of these same pathogens. What is not known is how effectively the alcohol-based hand sanitizers kill protists, fungi, or bacteria that cause eye disease. Here we show ethanol and isopropanol penetrate the walls and kill a free-living protist (*Acanthamoeba castellanii*, *A*. *byersi*, and other *Acanthamoeba spp*.), and a fungus (*Fusarium solani*), each of which causes keratitis, as well as a bacterium (*Chlamydia trachomatis*) that causes trachoma. These results suggest the possible benefit of hand sanitizers in the prevention of these eye pathogens.

## Introduction

*Acanthamoeba castellanii*, *A*. *byersi*, and other *Acanthamoeba spp*. are free-living protists named for actin-mediated spikes (acanthopods) on their surface that resemble those of acanthocytes (deformed red cells associated with various disease states) [1]. *Acanthamoeba* infections, which are often associated with use of contact lenses, may cause of corneal inflammation (keratitis) and blindness [2–4]. *Acanthamoebae* may also cause granulomatous encephalitis, particularly in immunosuppressed patients [5]. *Acanthamoebae* may host *Legionella pneumophilia*, *Vibrio cholera*, *Campylobacter jejuni*, and *Listeria monocytogenes* and so likely contribute to pneumonia, diarrhea, or disseminated disease caused by these pathogenic bacteria [6–9]. *Acanthamoebae* may contain enormous double-stranded DNA viruses (mimiviridae and Marseilleviridae), which may cause respiratory infections [10, 11]. In addition to mimivirus genomes, which contain nearly one thousand genes, the *Acanthamoeba* nucleus contains ~500 genes that were laterally transferred from bacteria (by far the most of any eukaryote described to date) [12].

The motile, reproductive, and vegetative form of *Acanthamoeba*, which has the acanthopods and phagocytoses bacteria, is called a trophozoite. When deprived of nutrients, trophozoites form cysts, which are immotile and have cellulose fibers in the wall [13]. During the course of keratitis infection, *Acanthamoeba* breaks down the epithelial barrier, induces an intense inflammatory response, and produces tears, photophobia, and pain due to radial neuritis [14]. Mouse and rabbit models of *Acanthamoeba* keratitis have identified protist virulence factors (e.g. mannose-binding lectin and secreted proteases) and host factors that mediate resistance to the protist (e.g. IL-17A) [15–17]. *Acanthamoeba* infections are treated with drug combinations (e.g. polyhexamethylene biguanide, chlorhexidine digluconate, propamidine isethionate, and hexamidine) [18]. These drugs are much more effective against trophozoites than cysts [19]. To date, efforts to prevent *Acanthamoeba* transmission have focused on the development of cleaning methods for contact lenses that kill trophozoites and cysts [20, 21]. Hand contamination with *Acanthamoeba* is likely also a significant contributor to its transmission.

Fungi that cause keratitis include *Candida albicans*, *Aspergillus fumigatus*, and *Fusarium solani*, the focus of studies here [22, 23]. *Fusarium* is a large genus of filamentous fungi that form septate hyaline hyphae about 3 to 8 microns in diameter. They also form fusoid macroconidia (multicellular, banana-like clusters), microconidia (unicellular, ovoid to cylindrical forms) and chlamydospores (dark, smooth-surfaced spheres). On culture plates *Fusarium* is identified on the basis of colony appearance, pigmentation, and growth rate [24]. *Fusarium* causes wilt in potatoes and root rot in tomatoes (*F*. *solani*), head blight in barley and wheat (*F*. *graminaerum*), ear rot in maize (*F*. *verticilloides*), and banana wilt (*F*. *oxysporum*) [25]. *F*. *sporotrichioides* produces a trichothecene mycotoxin, which may be an agent of biological warfare [26]. Fungal keratitis, which is an important cause of blindness in Asia, is associated with trauma by vegetable matter, as well as contact lens use (*Aspergillus* and *Fusarium*) or systemic disease (*Candida*) [27, 28]. *Fusarium* was also responsible for an outbreak of keratitis in the US associated with wearing soft hydrophilic contact lenses [29]. The infectious forms of *F*. *solani* are likely the conidia, which are asexually produced spores.

Fungal keratitis causes foreign body sensation and intense eye pain associated with excessive vascularization, epithelial defect, stromal infiltration, conjunctival hyperemia, hypopyon, and possible corneal perforation. Advanced keratitis can lead to endopthalmitis in immunocompromised, as well as immunocompetent individuals [30]. Since clinical presentation of fungal keratitis is inconclusive, cultures of corneal scraping, tissue biopsies, and PCR are frequently used for the definitive diagnosis of the infection [31]. Treatment is accomplished using different anti-mycotic agents including natamycin, amphotericin B, voriconazole, and ketoconazole [32–34]. Surgical approaches include keratoplasty and eye enucleation, in the case of treatment failure [35].

Because of the tremendous economic impact of *Fusarium* species on agriculture, much more is known about plant pathogenesis than human pathogenesis [36]. However, fluorescent labeled fungi have been used to study *F*. *solani* keratitis in mice [37], and spectral domain optical coherence tomography has been used to determine the order of events in a rat model of contact-lens associated keratitis [38].

Trachoma, which results from recurrent infections of the eye with the ocular serovars of the obligate intracellular bacterium *Chlamydia trachomatis*, is the leading infectious cause of blindness [39]. According to the World Health Organization (WHO), trachoma is responsible for the visual impairment of about 2.2 million people, of whom 1.2 million are irreversibly blind [40]. Trachoma has a significant social and economic impact in poor, rural areas of Africa, Asia, and the Middle East, which are least equipped to manage such infections. The replicating form *C*. *trachomatis* is the reticulate body (RB), which develops within in an intracytoplasmic vacuole of the host cell known as an inclusion. The RBs, however, are not infectious. Only the elementary body (EB) form, which is small and metabolically inactive, is capable of initiating infection.

Trachoma begins in childhood where repeated episodes of conjunctival infection with the ocular serovars of *C*. *trachomatis* leads to inflammation and scarring that, over time, results in trichiasis, where the eye lid contracts and the eyelashes turn inward where they cause trauma to the corneal surface. Eventually this leads to corneal opacification and blindness [39].

In order to eliminate blindness due to trachoma by 2020, the WHO endorsed the SAFE strategy through surgery, antibiotics, facial cleanliness, and environmental improvement [41–43]. Surgical procedures for treatment of trichiasis have recently been summarized [44]. Mass treatment of an entire community with azithromycin has been successful in decreasing the prevalence of trachoma, but it is expensive, treatment must be repeated, and it can lead to antibiotic resistance of co-circulating pathogens such as *Streptococcus pneumoniae* within a community [45]. Water, sanitation, and hygiene (WASH) are important components of facial cleanliness and hygiene in trachoma elimination strategies [46, 47]. Reduction in *Acanthamoeba* and *Fusarium* infections has also focused on hand washing with soap and water, as well as the best methods for cleaning contact lenses. A major limitation of these strategies is water scarcity, which faces four billion persons, primarily in Africa and Asia [48, 49].

The question addressed here is what might be done to prevent infections with *Acanthamoeba*, *F*. *solani*, and *C*. *trachomatis* in places where water for hand washing with soap is unavailable? In particular, might alcohol-based hand sanitizers, which are widely used to prevent spread of bacterial and fungal infections in hospitals in developing and developed countries, kill these important eye pathogens [50–59]? As a model for these studies, we recently showed that ethanol and isopropanol in concentrations present in hand sanitizers kill cysts of *Giardia* and *Entamoeba*, important causes of diarrhea and dysentery, respectively [60]. In each case, the alcohols penetrate the cyst walls and permeabilize the plasma membrane, so that nuclei stain with propidium iodide (PI), and trophozoites fail to excyst in culture or in animals (*Giardia*). Here we show that ethanol and isopropanol can kill trophozoites and cysts of *Acanthamoeba*, conidia of *F*. *solani*, or elementary bodies of *C*. *trachomatis*.

## Methods

### Ethics statement

Culture and manipulation of *Acanthamoeba*, *F*. *solani*, *and Chlamydia spp*. have been approved by the Boston University Institutional Biosafety Committee.

### *Acanthamoeba* methods

Trophozoites of *A*. *castellanii* MEEI 0184strain and *Acanthamoeba spp*. Esbc4 (PHLS) and Shi (PHLS) strains, each of which was derived from a human corneal infection, were generous gifts of Dr. Noorjahan Panjwani of Tufts University School of Medicine. Trophozoites were grown in PYG medium, which contains 0.75% proteose peptone, 0.75% yeast extract, and 1.5% glucose at 30°C (Sigma-Aldrich Corporation, St. Louis, MO) [61]. *Acanthamoeba byersi* was obtained from American type culture collection (ATCC PRA-411) and was grown in PYG medium containing 2% proteose peptone, 0.1% yeast extract and 1.8% glucose along with additives. Log-phase trophozoites, which were free of cysts that form spontaneously in stationary cultures, were concentrated by centrifugation at 3000 rpm and washed twice with phosphate buffered saline (PBS), which contained 10 mM PO_4_^3-^, 137 mM NaCl, and 2.7 mM KCl, pH7.4. Trophozoites (10^6^/ml in duplicate tubes) were suspended in water, 63% ethanol, or 63% isopropanol for 30, 60, or 120 sec at room temperature. After treatment, the trophozoite suspension was diluted x 10 with 1X PBS, pelleted at 3000 rpm for 10 min, washed x 2 in PBS, and cultured for three days in PYG medium. Trophozoites were counted using phase microscopy and a haemocytometer. Differential interference microscopy (DIC) was used to discriminate live organisms (motile with acanthopod spikes) from dead organisms (immotile and rounded). Alternatively, untreated and alcohol-treated trophozoites were incubated with Syto 9 (Thermo Fisher Scientific, Cambridge, MA), a vital stain that penetrates the plasma membrane, and PI, a nuclear stain that does not penetrate the plasma membrane [60, 62, 63]. Using a Zeiss inverted fluorescence microscope, we determined how many trophozoites were live (label green with Syto 9 but not red with PI) or dead (label with both Syto 9 and PI).

*Acanthamoeba* trophozoites were induced to encyst by incubation at 30°C for ten days on non-nutrient agar plates. Intact cysts (10^6^/ml), which were obtained by scraping plates, were incubated in water, 63% ethanol, 80% ethanol, 63% isopropanol, or 80% isopropanol for 30, 60, or 120 sec at room temperature, diluted x 10 with PBS, pelleted at 3000 rpm for 10 min, and washed x 2 in PBS to remove alcohols. To simulate evaporation of alcohols on hands, we dried some cysts with a rotary vacuum after removal of alcohols. Cysts +/- drying were placed in PYG medium for 24 hours at 30°C to measure excystation of trophozoites. Alternatively, untreated and alcohol-treated amoebae were labeled with PI and wheat germ agglutinin (WGA) (Sigma-Aldrich), which binds to glycoproteins in the cyst wall. Fluorescence microscopy was used to discriminate live cysts (label with WGA but not PI) from dead cysts (label with both WGA and PI).

### *Fusarium* methods

*F*. *solani* (ATCC 36031) was purchased from American Type Culture Collection (Manassas, Virginia) and grown on a potato dextrose agar plate (BD 213400) (Becton, Dickinson and Company, Franklin Lakes, NJ) for 48 hr at 30°C. Conidia were removed from the agar plate with a cell scraper, filtered through a gauze pad, and washed with PBS x 2 by centrifugation at 3000 rpm for 10 min. *F*. *solani* conidia (1x10^6^/ml) were resuspended in water, 63% ethanol, or 63% isopropanol for 30, 60, or 120 sec at room temperature, diluted X 10 with PBS, and then pelleted at 3000 rpm for 10 min, and resuspended in water for plating. Conidia were serially diluted, and ~10 or ~100 conidia were plated on potato dextrose agar plate from control tubes, whereas one million conidia were inoculated from ethanol-treated and isopropanol-treated tubes. Alternatively, conidia were labeled with PI and WGA, which binds to chitin in fungal walls, and observed with a Zeiss fluorescence microscope to determine live (labels with WGA but not PI) and dead (labels with both WGA and PI).

### *Chlamydia* methods

The ocular serovar *C*. *trachomatis* strain A2497, originally isolated from a 2.5 year old with intense active trachoma in Tanzania, has been sequenced and used in non-human primate studies of trachoma pathogenesis and vaccination [64, 65]. Gradient purified EBs were prepared and titered as previously described [66]. For each experimental condition, *C*. *trachomatis* EBs suspended in sucrose-phosphate-glutamic acid (SPG) buffer were added to ethanol or isopropanol to achieve a final alcohol concentration of 63% and allowed to incubate at room temperature for 30 or 60 sec. Mock treated EBs were diluted in SPG alone for the same time period. The alcohol was not allowed to evaporate on the EBs. At the end of the incubation period, the alcohol was diluted with chilled RPMI-1640 medium (Thermo Fisher Scientific)) containing 10% low endotoxin fetal bovine serum (FBS) (HyClone Laboratories, Logan UT), gentamicin (20 μg/ml) and cycloheximide (2 μg/ml), and EBs were inoculated in triplicate to a monolayer of L929 fibroblasts (ATCC) plated in the same medium in 96-well or 24-well tissue culture dishes at a multiplicity of infection (MOI) of 1:10. The final alcohol concentration on the monolayer was less than 0.5%; control wells were inoculated with the same final dilution of alcohol to assess for cytotoxicity to the monolayer. After 44 hr incubation at 37%/5% CO_2_, cells were fixed in methanol and EBs were stained using a *Chlamydia*-specific LPS monoclonal antibody (gift of Dr. You-Xun Zhang of Boston Medical Center), followed by FITC-conjugated secondary antibody. Cells were counter stained with Evans blue (Sigma-Aldrich). Antibodies were used at a concentration of 10 μg/ml and incubated for 20 min with washing in between each step. The inclusions were counted under fluorescent microscopy, and the number of infectious units recovered per well was determined. Percent killing was calculated based on the inclusions recovered from mock treated EBs.

## Results

### Ethanol and isopropanol kill trophozoites of *Acanthamoeba* in 30 seconds

**Fig 1:**
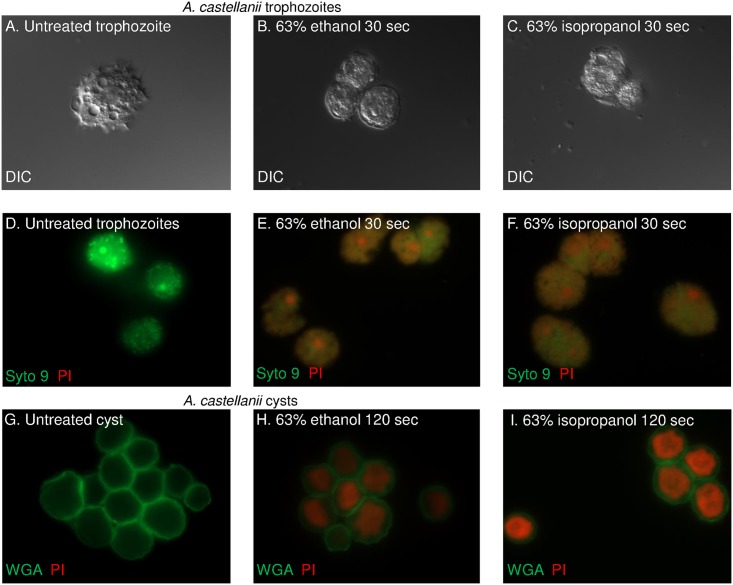
*A*. *castellanii* trophozoites and cysts are rapidly permeabilized and killed by ethanol and isopropanol. (A) Differential interference contrast (DIC) micrograph shows that untreated trophozoites have prominent vacuoles and acanthopods. In contrast, trophozoites treated with 63% ethanol (B) or 63% isopropanol (C) for 30 sec are rounded and have lost acanthopods. Not captured in still photographs is mobility of untreated trophozoites versus absence of mobility of alcohol-treated trophozoites. (D) Fluorescence micrograph of untreated trophozoites shows that Syto 9 (green), a vital stain, readily penetrates the plasma membrane, while PI (red stain in live-dead kits) does not. In contrast, both Syto 9 and PI readily label trophozoites killed by incubation in ethanol (E) and isopropanol (F). Wheat germ agglutinin (WGA in green) labels glycoproteins in the walls of both untreated (G) and alcohol-treated cysts for 120 sec (H and I). PI (red) does not label untreated cysts but readily penetrates walls and plasma membrane of alcohol-treated cysts. All images are of *A*. *castellanii* MEI 0184 strain and were shot through the 100X objective.

**Fig 2:**
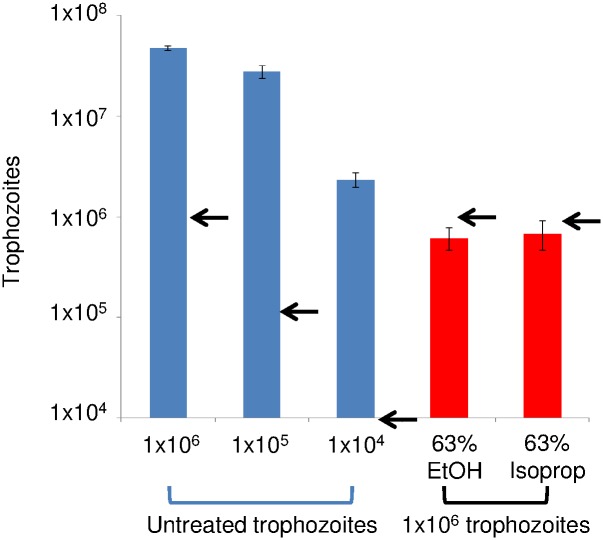
Treatment with alcohols blocks all growth of *A*. *castellanii* trophozoites in culture. Log plots of growth in culture shows untreated trophozoites (blue), which were undiluted or diluted 10-fold or 100-fold, increase their number by 23 to 47 times in culture for three days. In contrast, ethanol-treated and isopropanol-treated trophozoites (red) show no growth in culture, and all of the remaining trophozoites are dead (as judged by either lack of mobility and lack of acanthopods by DIC or PI labeling by fluorescence microscopy) (previous figure). Plots shown are for *A*. *castellanii* MEI 0184 strain, but the same results were obtained with the other *Acanthamoeba spp*. strains and with *A*. *byersi*. Arrows indicate number of trophozoites at start of incubation.

The motile, dividing form of *Acanthamoeba*, is the trophozoite, while the immotile, non-dividing and walled form is the cyst. Because both trophozoites and cysts are capable of causing eye infections, we tested ethanol and isopropanol against both forms. We found that trophozoites of *A*. *castellanii* MEI 0184 strain, *Acanthamoeba spp*. Shi and Esbc4 strains, and an *A*. *byersi* isolate are each rapidly killed (within 30 sec) by exposure to 63% ethanol or 63% isopropanol, as shown by loss of motility and acanthopods (DIC microscopy), as well as permeability of plasma membranes to PI (red in fluorescence microscopy) (Fig 1A to 1F). Syto 9 (green in fluorescence microscopy) is a vital stain that penetrates through intact plasma membranes [47]. While untreated trophozoites rapidly multiply in culture for three days, there is no growth of alcohol-treated *Acanthamoebae*, and all those recovered are dead (as shown by DIC and/or fluorescence microscopy) (Fig 2).

### Longer times or higher concentrations of ethanol are needed to kill *Acanthamoeba* cysts

**Fig 3:**
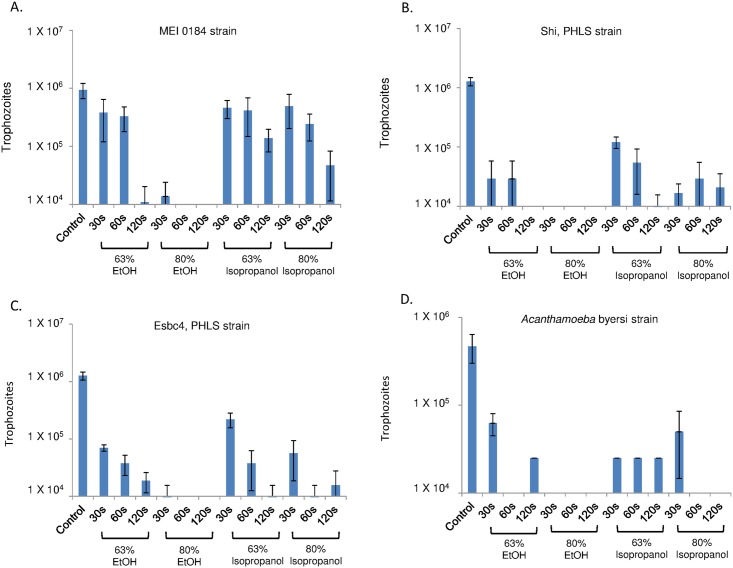
Treatment of *Acanthamoeba* cysts with alcohols reduces excystation and trophozoite growth. Log plots of excystation and growth of *A*. *castellanii* MEI 0184 strain (A), *Acanthamoeba spp*. Shi (B) and Esbc4 (C) strains, and *A*. *byersi* (D). Compared to killing of trophozoites (Fig 2), killing of cysts was much slower with 63% ethanol and much less effective with isopropanol. However, all four sets of *Acanthamoeba* cysts were rapidly killed by 80% ethanol.

Cysts of *Acanthamoeba* are also killed by exposure to ethanol and isopropanol, as shown by permeability of walls and plasma membrane to PI and by reduced excystation when placed in culture medium (Figs 1G to 1I and 3A to 3D). Here there was considerable variability among the four *Acanthamoeba* strains examined. In each case, though, it took longer to kill cysts in 63% ethanol (up to 120 sec), although all four sets of cysts were killed in 30 sec in 80% ethanol (the upper limit for alcohol in hand sanitizers). Isopropanol was much less effective than ethanol in killing *Acanthamoeba* cysts. Further, drying of cysts in a rotary evaporator had no effect on excystation.

### Both alcohols kill conidia of *F*. *solani* in 30 seconds

**Fig 4:**
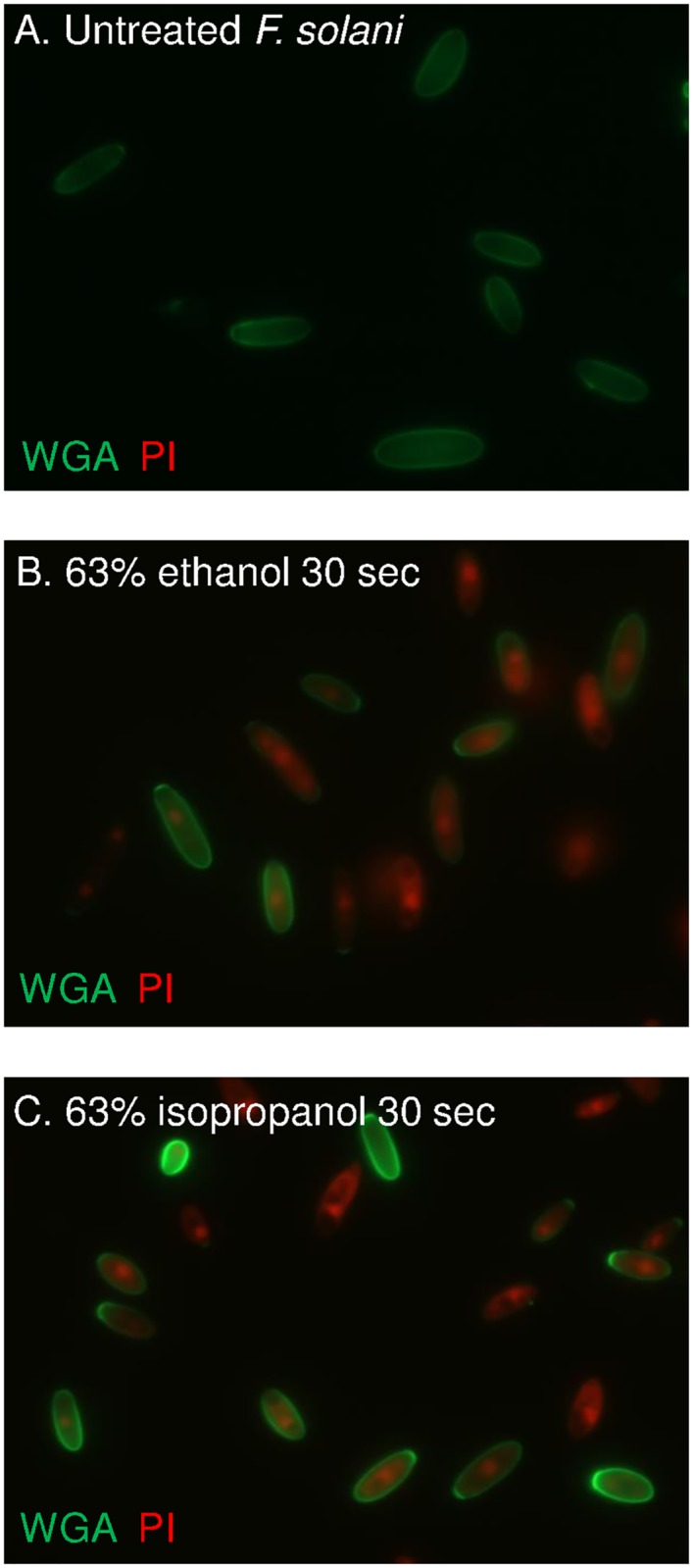
*F*. *solani* conidia are rapidly permeabilized and killed by ethanol and isopropanol. WGA (green) labels glycoproteins in the walls of both untreated and alcohol-treated conidia (A to C). In contrast, PI (red) fails to penetrate walls of untreated conidia (A), while PI readily penetrate walls and plasma membrane and labels nuclei of alcohol-treated conidia for 30 sec (B and C). All images shot through the 100X objective.

**Fig 5:**
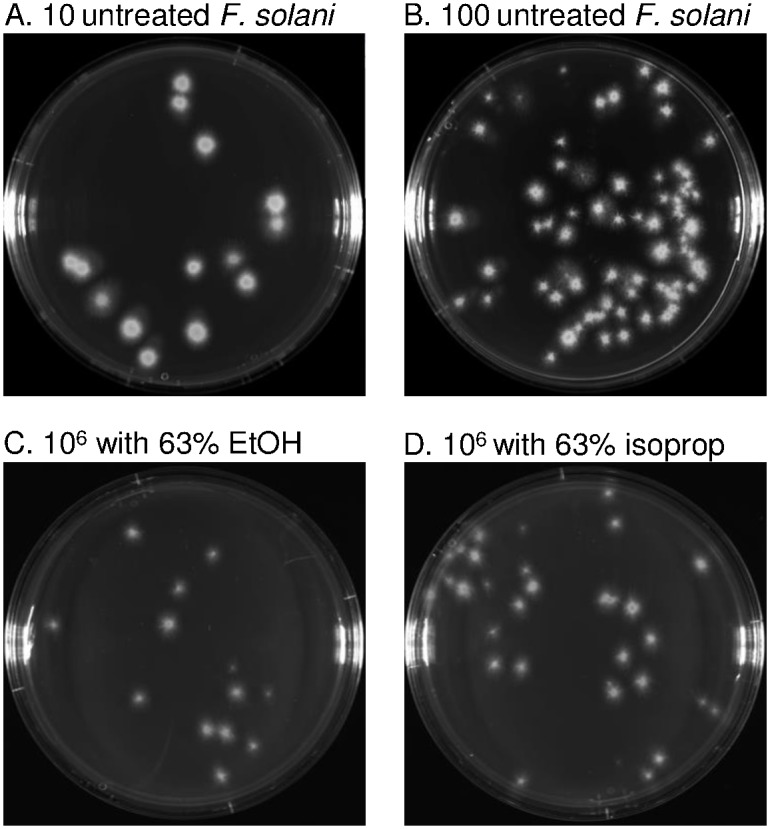
Treatment of *F*. *solani* conidia with ethanol and isopropanol markedly inhibits colony formation on nutrient agar plates. Ten (A) or 100 (B) untreated *F*. *solani* conidia form discrete colonies on nutrient agar plates that may be counted. When 10^6^ conidia treated for 30 sec with ethanol (C) or isopropanol (D), diluted, pelleted, washed, and then plated, nearly the same number of colonies appear as when 10 untreated conidia are plated.

*F*. *solani* conidia, which were grown on potato dextrose agar plates, are killed within 30 sec by 63% ethanol or 63% isopropanol, as shown by permeability of walls and plasma membrane to PI and by colony counting on culture plates (Figs 4A to 4C and 5A to 5D). For the latter, conidia were treated with alcohols, diluted, pelleted, washed, and then plated at various dilutions. We counted nearly the same colonies from 10 untreated *F*. *solani* conidia as from 10^6^ ethanol-treated or isopropanol-treated conidia. These results suggest that >99% of the *F*. *solani* conidia are killed by each alcohol.

### Alcohols inactivate EB of *C*. *trachomatis* in 60 seconds

**Fig 6:**
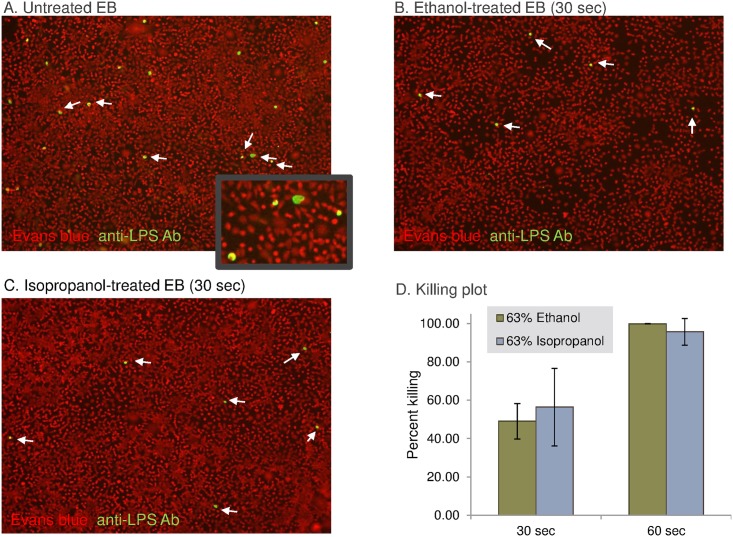
Elementary bodies of *C*. *trachomatis* are killed by ethanol and isopropanol. (A) Fluorescence micrograph of a monolayer of L929 fibroblasts infected at an MOI of 1:10 with mock-treated *C*. *trachomatis* ocular serovar A2497 EBs. *C*. *trachomatis* inclusions stain green (arrows), while L929 cells are counterstained red. In contrast, after exposure for 30 sec to 63% ethanol (B) or 63% isopropanol (C), the development of inclusions is markedly reduced. After 60 sec treatment, few to no inclusions were visible in the wells. Images shot through the 10X objective. (D) Quantitative cultures of the alcohol treated EBs were compared to the mock treated EBs to calculate the percent killing. Shown above are the mean +/- standard deviation of triplicate wells. Data is representative of three independent experiments.

Because *C*. *trachomatis* is an obligate intracellular bacterium, we modified our approach so that purified chlamydial EBs, the infectious forms of the organism, were incubated with 63% ethanol or 63% isopropanol for 30 or 60 sec. We then diluted the alcohol exposed EBs and inoculated them onto L929 fibroblasts to determine if they were capable of developing intracellular inclusions, since at a low MOI each inclusion can be assumed to grow from one EB. Treatment of EBs with ethanol (Fig 6B) or isopropanol (Fig 6C) for 30 sec prior to plating markedly reduced the number of recovered inclusions compared to mock treated EBs (Fig 6A). However, after a 60 sec exposure to alcohol, extremely rare or no inclusions were visible in either the ethanol- or isopropanol-treated wells. These data are quantified in Fig 6D, demonstrating approximately 50% killing of the EBs after 30 sec and 96–99% killing after 60 sec exposure. In summary, killing of EB of *C*. *trachomatis* by 63% ethanol takes longer (60 sec) than killing conidia of *F*. *solani* (30 sec) but takes shorter time than killing *Acanthamoeba* cysts (120 sec).

## Discussion

### Pros of alcohol-based hand sanitizers to reduce infections with diverse eye pathogens

Treatment with ethanol or isopropanol in the concentrations present in hand sanitizers kills *Acanthamoeba* trophozoites, *F*. *solarium* conidia, and *C*. *trachomatis* elementary bodies (Figs 3, 5 and 6). In contrast, longer times or higher ethanol concentrations are necessary to kill cysts of *Acanthamoeba*, and isopropanol is much less effective at killing cysts. Labeling with PI shows that the walls and membranes of *Acanthamoeba* and *F*. *solani* are penetrated by the alcohols (Figs 1 and 4), as has been shown from cysts of *Giardia* and *Entamoeba* [60]. Although the small size makes it difficult to study the cell wall architecture, it is likely that the bacterial membranes of the chlamydial EBs are also penetrated by alcohols. Drying, which occurs when alcohols evaporate from the hands, was not necessary for alcohol-based killing of these organisms. These results extend previous findings showing that treatment of *Acanthamoeba* cysts with alcohols and other organic solvents for 10 min reduces excystation, while numerous additives to contact lens solutions prevents the growth of contaminating *Acanthamoeba* trophozoites [67–70].

Alcohol-based hand sanitizers are safe, relatively inexpensive, stable at room temperature, and do not select for antibiotic resistance. Hand sanitizers also reduce infections with viruses, bacteria, and parasites that cause diarrhea [50, 52–59], as well as fungi that may cause systemic infections [51]. It appears then that alcohol-based hand sanitizers, particularly those with high concentrations of ethanol, might join the armamentarium of preventative efforts against the representative ocular pathogens tested here, particularly where water is scarce, so that hand washing with soap is not possible. In the case of *C*. *trachomatis*, hand sanitizers might contribute to the facial cleanliness arm of the WHO’s SAFE strategy to combat trachoma [43].

### Cons of alcohol-based hand sanitizers

Alcohols do not treat current eye infections, which are treated with antibiotics +/- surgery. Because of the potential of alcohols to damage the corneal epithelium, they cannot be used to wash eyes or to wash hands immediately before handling contact lenses. As cysts, conidia, and elementary bodies were treated with alcohols while present in test tubes rather than on the human skin, it is possible that the efficiency of killing maybe less when hands are washed with hand sanitizers. Consistent with the experience of hand washing with soap and water, efficacy of hand sanitizers likely varies dramatically with thoroughness and time spent washing hands, as well as its consistency. Drying effects of alcohols, particularly when used in high concentrations, may also reduce their routine use. Finally, because the eye pathology caused by infections with *Acanthamoeba*, *F*. *solarium*, and *C*. *trachomatis* occurs on a long time scale, it may be difficult to measure the efficacy of introducing hand sanitizers to a particular community. Despite these limitations, there may be role of alcohol-based hand sanitizers, which rapidly kill infectious forms of *Acanthamoeba*, *F*. *solarium*, and *C*. *trachomatis*, in reducing transmission of these important eye pathogens.
